# Effect of different disinfectants on preventing asymptomatic bacteriuria and catheter-related urinary tract infection: a network meta-analysis

**DOI:** 10.3389/fruro.2023.1173885

**Published:** 2023-10-13

**Authors:** Zhi Hong Sun, Xue Wei Ma, Wei Sun, Ying Ji Wei, Yi Zhen Li, Dan Wang, Chang Xi Zhou, Guo Gang Xu, Gui Zhi Zhang

**Affiliations:** ^1^ Department of Pulmonary and Critical Care Medicine of the Second Medical Center & National Clinical Research Center for Geriatric Diseases, Chinese PLA General Hospital, Bejing, China; ^2^ Department of Geriatrics of the Second Medical Center & National Clinical Research Center for Geriatric Diseases, Chinese People's Liberation Army (PLA) General Hospital, Bejing, China; ^3^ Department of Health Medicine of the Second Medical Center & National Clinical Research Center for Geriatric Diseases, Chinese PLA General Hospital, Bejing, China

**Keywords:** catheter-related urinary tract infection, chlorhexidine, iodophor, acidic oxidizing potential water, meta-analysis

## Abstract

**Objective:**

To analyze the effect of different types of disinfectants for perineum and urethral meatus cleaning in preventing catheter-associated asymptomatic bacteriuria and catheter-associated urinary tract infection (CAUTI).

**Methods:**

Chinese and English databases were searched to collect randomized controlled trials (RCTs) of different disinfectants for perineum and urethral meatus cleaning to prevent CAUTI, and the positive rates of urine culture with different cleaning methods were contrasted by network meta-analysis.

**Results:**

A total of 18 RCTs were included in this study to analyze the effect of 10 disinfectants in preventing CAUTI. The disinfectants were 0.1% chlorhexidine (CHG), 0.5% CHG, 2% CHG, 0.1% benzalkonium bromide (DBDAB), 0.05% iodophor, 0.5% iodophor, acidic oxidizing potential water (EOW), normal saline (NS), Shehuang lotion, and sterile water. Meta-analysis showed that the positive rates of urine culture in the 0.5% CHG cluster, EOW cluster, 0.5% iodophor cluster, and Shehuang lotion cluster were remarkably lower than that in the 0.1% DBDAB cluster (P < 0.05). The positive rate of urine culture in the 0.5% CHG cluster was remarkably lower than that in the 0.05% iodophor cluster (P < 0.05), whereas the positive rates of urine culture in the 0.5% CHG cluster, 0.5% iodophor cluster, Shehuang lotion cluster, and EOW were remarkably lower than that in the normal saline cluster (P < 0.05). The positive rate of urine culture in the 0.5% CHG cluster and EOW cluster were remarkably lower than those in the sterile water cluster (P < 0.05). The results of the area under the cumulative ranking probability plot (SUCRA) analysis showed that the probability ranking of the preventive effect of different disinfectants was as follows: 0.5% CHG > Shehuang lotion > EOW > 0.5% Iodophor > 2% CHG > 0.1% CHG > 0.05% Iodophor > Sterile Water > 0.1% DBDAB > Saline.

**Conclusion:**

0.5% CHG, Shehuang lotion, EOW, and 0.5% iodophor can be used to clean the perineum and urethral meatus in patients with indwelling catheters.

**Systematic review registration:**

Chinese Clinical Trial Registry (ChiCTR), identifier ChiCTR2100052260.

## 1 Introduction

Catheter-associated urinary tract infection (CAUTI) refers to urinary tract infection that occurs between 48 hours after indwelling catheter and 48 hours after catheter removal and is an important component of nosocomial infection, accounting for more than 30% of nosocomial infection cases (1). CAUTI occurs in more than 3% of hospitalized patients and reaches 17. 6% in intensive care unit patients (2, 3). CAUTI can increase the risk of patient death, increase the use of antibacterial drugs, and prolong the length of hospital stay (4). Studies have shown that the mortality rate of CAUTI reaches 2.3% and rises to 9% when bacteremia occurs, with the mortality rate reaching 25% to 60% in patients who develop sepsis (5). Therefore, CAUTI should be actively prevented in clinical practice in patients who undergo urinary catheter indwelling, especially in patients with longer indwelling time and upper risk of infection. Periurethral colonization is an important source of CAUTI, so reducing bacterial colonization of the urethral meatus and surrounding area can reduce the risk of CAUTI (3). At present, a number of studies at home and abroad have investigated the effect of urethral meatus cleaning and perineal cleaning in the prevention of CAUTI, but there is no consensus on which disinfectant has the best effect. This study intends to investigate the effect of different types of disinfectants in preventing CAUTI through network meta-analysis, providing a reference for the selection of cleaning disinfectants for perineum and urethral meatus in patients with indwelling urinary catheters.

## 2 Materials and methods

### 2.1 Literature inclusion criteria

The study was approved by the ethics institutional review board of the General Hospital of the Chinese People’s Liberation Army. We searched PubMed, Embase, Medline (via Ovid SP), and Cochrane Library databases from January 2005 to December 2021. Sources of data in both Chinese and English were scanned, including PubMed, Web of Science, Embase, SinoMed, and Cochrane Library for English libraries and the Chinese Journal Full-text Database (CNKI), Wanfang Database, and VIP Database for Chinese datasets. The keywords used in the search were the following: urinary catheter-related urinary tract infection, disinfectant, prevention, chlorhexidine, iodophor, and randomized trial. English search terms were: catheter-associated urinary tract infection, disinfectant, chlorhexidine, iodophor, prevent/prevent, and randomized. MseSH-related terms were searched with subject terms combined with free terms.

### 2.2 Literature inclusion criteria

#### 2.2.1 Study subjects

Hospitalized patients with indwelling catheters.

#### 2.2.2 Study design

Randomized controlled trials (RCTs) of different disinfectants for perineum and urinary meatus cleaning to prevent CAUTI.

#### 2.2.3 Intervention

Perineum and urethral meatus cleaning was performed using the following disinfectants: 0.1% chlorhexidine (CHG), 0.5% CHG, 2% CHG, 0.1% benzalkonium bromide (DBDAB), 0.05% iodophor, 0.5% iodophor, acidic oxidizing potential water (EOW), normal saline (NS), Shehuang lotion, and sterile water.

#### 2.2.4 Outcome measures were reported

Positive urine culture rate.

### 2.3 Literature exclusion criteria

(1) The study with the broadest sample size included recurring findings for the same study participants.
(2) The antiseptics composition was unclear or could not be defined because cleaning agents were combined.
(3) The data seem to have been inadequate.
(4) The publications were posted in languages other than Chinese or English.
(5) Access to the full transcript was not possible.
(6) Studies including mouse models and evidence from animal models.
(7) The study was of inferior quality (Jadad < 3 points).

### 2.4 Data Extraction

Two research personnel searched databases independently for a comprehensive search. We deleted duplicate records, screened the titles and abstracts for relevance, and identified each as excluded or requiring further assessment. We reviewed the full-text articles designated for inclusion and manually checked the references of the retrieved articles and previous reviews to identify additional eligible studies. Discrepancies were resolved by consensus. The following data were extracted from each study: study design, first author, year of publication, sample size, positive urine culture, interventions, comparisons, and outcomes. A third researcher was consulted in case of disagreement between the two investigators.

### 2.5 Assessment of literature quality

The assessment of literature quality for each study was based on the modified Jadad scale (6). This scale has a maximum score of 7 and includes four parameters: random sequence generation, allocation concealment, intervention blinding of participants and researchers, and incomplete outcome data. Low and high-quality studies are indicated by Jadad scores of ≤ 3.0 and > 3.0, respectively.

### 2.6 Analysis methods

Data analysis was performed using Stata15 software. The odds ratio (OR) was used as the effect statistics for the healing rate and 95% CI was calculated. The agreement between direct evidence and indirect evidence was contrasted by the node-cutting method, and the results of consistency assessment in this study showed P > 0.05, and the consistency model was selected for analysis. Bayesian network model was used to calculate the ranking probability of CAUTI prevention by different disinfectants, and the possibility of each disinfectant becoming the best choice was presented by calculating the area under the cumulative ranking probability plot (SUCRA). Funnel plots were used to analyze the presence of publication bias.

## 3 Results

### 3.1 Literature screening process and basic characteristics of the included literature

**Figure 1:**
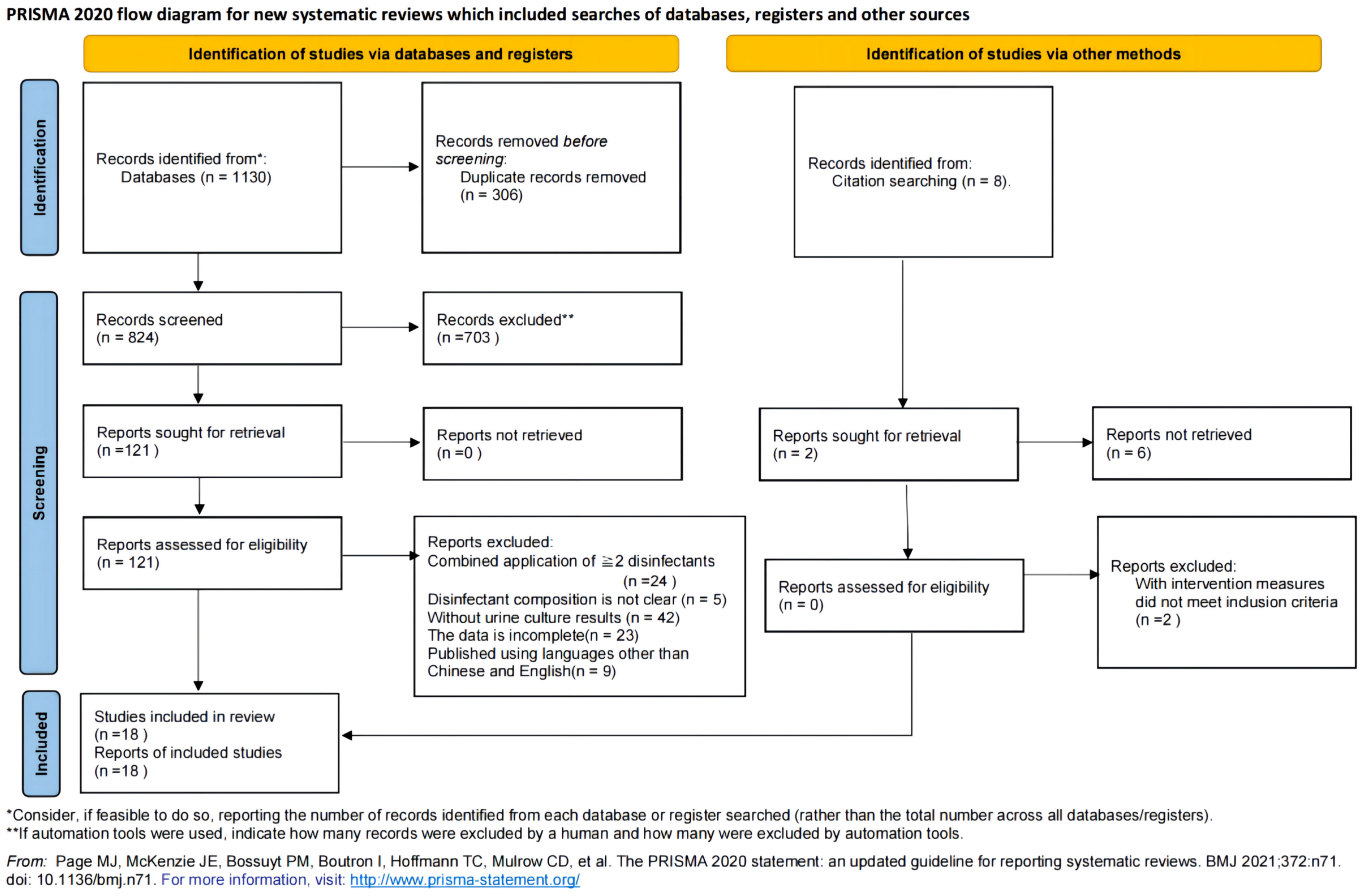
Literature screening procedure.

A total of 18 studies were included in this review (**Figure 1**). There were 14 studies in Chinese and 4 studies in English, with a total of 5,368 cases divided as follows: 2,722 cases in the test cluster (cluster T) and 2,646 cases in the control cluster (cluster C). There were 13 studies with Jadad scores of 3 ~ 7 points and ≥ 4 points, and 72.2% of them were high-quality studies (**Table 1**).

**Table 1:** Basic characteristics of included studies.

Author	Year	Positive urine culture/sample size (T)	Positive urine culture/sample size (C)	Disinfectant		Jadad
				T	C	
Xu B (7)	2006	5/30	7/32	0.5% CHG	Sterilized water	3
Leng XH (8)	2008	11/59	9/61	0.05% Iodophor	Sterilized water	3
Shen Y (9)	2008	5/40	17/40	0.5% Iodophor	NS	3
He SP (10)	2010	7/50	12/50	0.5% Iodophor	0.1% DBDAB	4
Wu XX (11)	2015	8/42	16/42	0.5% CHG	Sterilized water	6
Liu JM (12)	2011	12/60	37/60	0.5% Iodophor	0.1% DBDAB	3
Zheng XY (13)	2016	9/46	23/46	0.5% Iodophor	Sterilized water	7
Li CX (14)	2014	9/50	8/50	EOW	0.5% Iodophor	6
Chen CQ (15)	2017	11/105	18/108	Shehuang lotion	0.5% Iodophor	5
Luo Y (16)	2015	12/60	16/60	EOW	0.5% Iodophor	6
Bai SZ (17)	2016	9/60	16/60	EOW	0.5% Iodophor	4
Xia Y (18)	2021	6/96	54/100	0.5% CHG	Sterilized water	6
Fasugba O (19)	2019	20/945	42/697	0.1% CHG	NS	7
Cheung K (20)	2008	8/12	7/8	0.5% CHG	Sterilized water	5
Popovich KJ (21)	2009	23/561	47/672	2% CHG	Sterilized water	7
Bleasdale SC (22)	2007	44/445	4/55	2% CHG	Sterilized water	7
Song SK (23)	2015	4/55	10/55	0.05% Iodophor	NS	3
Chen XH (24)	2016	15/60	28/60	EOW	Sterilized water	4

### 3.2 Meta-analysis results

#### 3.2.1 Evidence network of CAUTI prevention by different disinfectants

**Figure 2:**
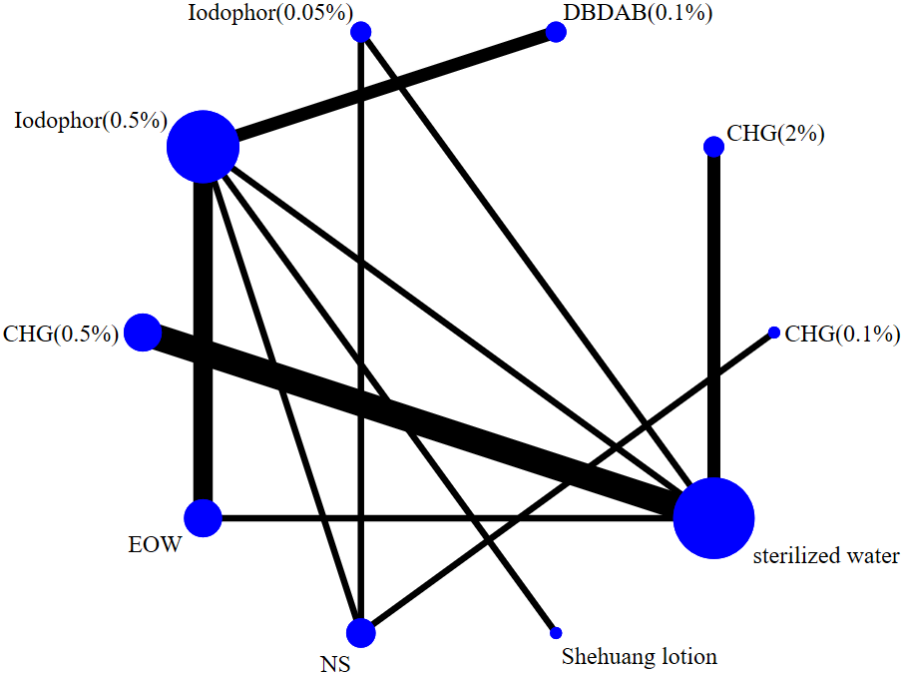
Network diagram of evidence for CAUTI prevention by different disinfectants.

This analysis compared the preventive effect of 10 disinfectants on CAUTI. The network relationship diagram of different disinfectants is shown in **Figure 2**. The nodes and edges (connections) show both direct and indirect comparisons. The size of the bubble and the thickness of the connections indicate the size and strength of head-to-head comparisons. It can be seen that the comparison between sterile water and 0.5% iodophor is the most common in this analysis.

#### 3.2.2 Comparisons between direct and indirect evidence

In cases where a loop connected three intersecting disinfectants, the node-splitting method was used to calculate the inconsistency of the model. The method separated the evidence concerning certain comparisons into direct and indirect evidence, and the inconsistency was reported by its Bayesian P value. For the majority of our results, most of the P values from the node-splitting method were above 0.05.

Node analysis of consistent results showed that there was no remarkable variance in the positive rate of urine culture between direct comparisons and indirect comparisons (P > 0. 05), and consistent model analysis could be used (**Table 2**).

**Table 2:** Consistency of direct comparisons and indirect comparisons in node analysis of positive rate of urine culture with different disinfectants.

Comparative Cluster	Direct Comparison		Indirect Comparison		*P value*
	Coef	Se	Coef	Se	
A vs. H	1.087	0.610	1.415	18.923	0.986
B vs. J	0.477	0.425	0.457	174.502	1.000
C vs. E	-1.320	0.509	-1. 411	100.600	0.999
D vs. H	1.041	0.860	0.304	1.257	0.628
D vs. J	-0.281	0.768	0.457	1.314	0.628
E vs. G	-0.336	0.439	0.206	0.977	0.613
E vs. H	1.643	0.823	2.378	1.279	0.629
E vs. I	-0.536	0.681	1.483	184.464	0.991
E vs. J	1.414	0.735	0.550	0.697	0.394
F vs. J	1.488	0.422	0.457	72.576	0.989
G vs. J	0.965	0.712	1.507	0.801	0.613

A, 0. 1 CHG; B, 2% CHG; C, 0. 1% DBDAB; D, 0. 05% Iodophor; E, 0. 5% Iodophor; F, 0. 5% CHG; G, EOW; H, NS; I, Shehuang lotion; J, Sterilized water.

#### 3.2.3 Meta-analysis of the effect of different disinfectants in preventing CAUTI

**Figure 3:**
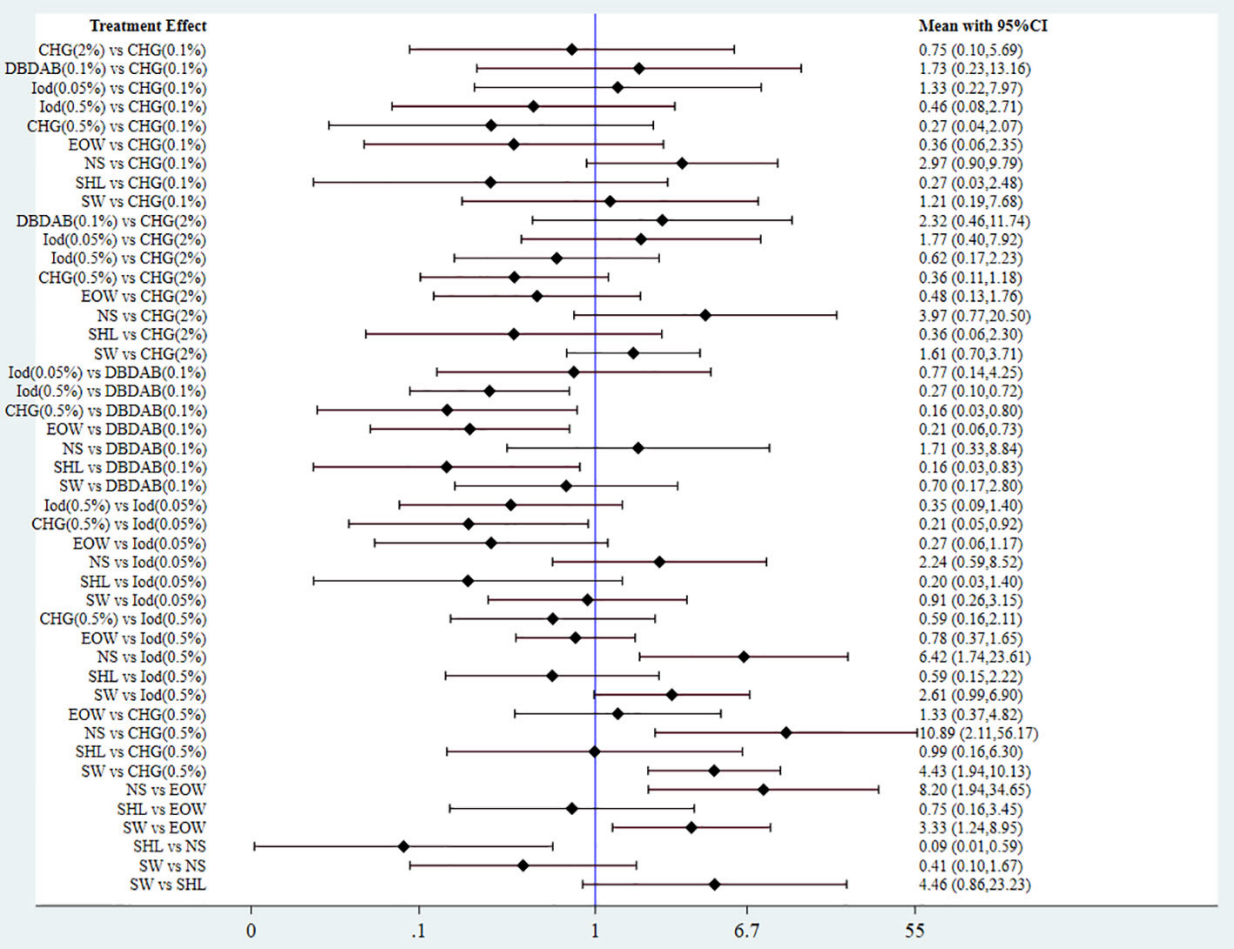
Forest map of meta-analysis of the effect of different disinfectants in preventing CAUTI. SHL, shehuang Lotion; Iod, Iodophor; SW, sterilized water.

A comparison of positive rates of urine culture with the use of different disinfectants is shown in **Figure 3**. The positive rate of urine culture in the 0.5% CHG cluster, EOW cluster, 0.5% iodophor cluster, and Shehuang lotion cluster was remarkably lower than that in the 0.1% DBDAB cluster (P < 0.05). The positive rate of urine culture in the 0.5% CHG cluster was remarkably lower than that in 0.05% iodophor cluster (P < 0.05), whereas the positive rates of urine culture in the 0.5% CHG cluster, 0.5% iodophor cluster, Shehuang lotion cluster, and EOW were remarkably lower than those in the normal saline cluster (P < 0.05). The positive rate of urine culture in the 0.5% CHG cluster and EOW cluster were remarkably lower than those in the sterile water cluster (P < 0. 05), and no variance was registered between the other two clusters (P > 0.05).

#### 3.2.4 Probability ranking and SUCRA ranking

**Figure 4:**
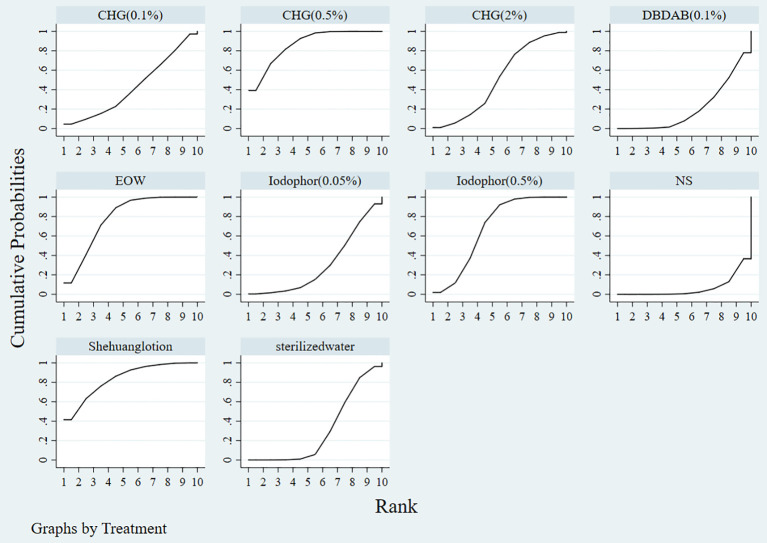
SUCRA Diagram of the Effect of different Disinfectants in Preventing Catheter-associated asymptomatic bacteriuria and CAUTI.

The probability ranking of different types of disinfectants in preventing catheter-associated asymptomatic bacteriuria and CAUTI is displayed in **Table 3**. Shehuang lotion has the highest probability of preventing catheter-associated asymptomatic bacteriuria and CAUTI, with a probability ranking of 41.5%, followed by 0.5% CHG (39.2%); 0.5% CHG has the highest probability of ranking second, 27.7%; EOW has the highest probability of ranking third, 30. 4%; and normal saline has the worst probability of preventing catheter-associated asymptomatic bacteriuria and CAUTI, 63.4%. The ranking results of SUCRA are shown in **Table 4**; the larger the SUCRA, the better the effect of preventing catheter-associated asymptomatic bacteriuria and CAUTI. The ranking, from high to low, was as follows: 0.5% CHG > Shehuang Lotion > EOW > 0.5% iodophor > 2% CHG > 0.1% CHG > 0.05% iodophor > sterile water > 0.1% DBDAB > normal saline. SUCRA plots are shown in **Figure 4**.

**Table 3:** Probability ranking of CAUTI prevention effect by different types of disinfectants (%).

Sort	SHL	0.5%CHG	EOW	0.5%Iod	2%CHG	SW	0.05%Iod	0.1%CHG	0.1% DBDAB	NS
Best	41.5	39.2	11.6	1.9	1.0	0.0	0.4	4.5	0.0	0.0
2^nd^	21.8	27.7	26.3	9.9	4.8	0.0	1.2	5.2	0.1	0.0
3^rd^	12.9	14.7	30.4	25.4	8.4	0.2	1.8	5.8	0.3	0.0
4^th^	10.1	11.0	17.7	36.8	11.7	0.8	3.4	7.3	1.0	0.1
5^th^	6.4	5.7	10.8	18.3	27.8	4.7	8.5	14.1	6.3	0.4
6^th^	3.6	1.4	2.1	5.8	22.6	21.6	14.4	14.7	10.2	1.6
7^th^	2.0	0.2	1.0	1.7	12.3	32.0	20.8	13.9	14.4	3.7
8^th^	1.3	0.1	0.1	0.3	6.7	25.5	24.1	15.0	19.8	7.1
9^th^	0.3	0.0	0.0	0.0	3.5	11.5	18.4	16.8	25.8	23.7
Worst	0.1	0.0	0.0	0.3	1.1	3.7	7.0	2.7	22.0	63.4

SHL, Shehuang Lotion; Iod, Iodophor; SW, Sterilized water.

**Table 4:** SUCRA ranking of catheter-associated asymptomatic bacteriuria and CAUTI prevention effects of different disinfectants.

Disinfectant	SUCRA	Probability of best prevention (%)	Average ranking
0.5% CHG	86.5	39.2	2.2
SHL	83.8	41.5	2.5
EOW	78.7	11.6	2.9
0.5% Iod	68.2	1.8	3.9
2% CHG	51.1	1.0	5.4
0.1% CHG	42.7	4.5	6.2
0.05% Iod	30.7	0.4	7.2
SW	30.6	0.0	7.2
0.1% DBDAB	21.2	0.0	8.1
NS	6.5	0.0	9.4

SHL, Shehuang Lotion; Iod, Iodophor; SW, Sterilized water.

### 3.3 Risk of bias

**Figure 5:**
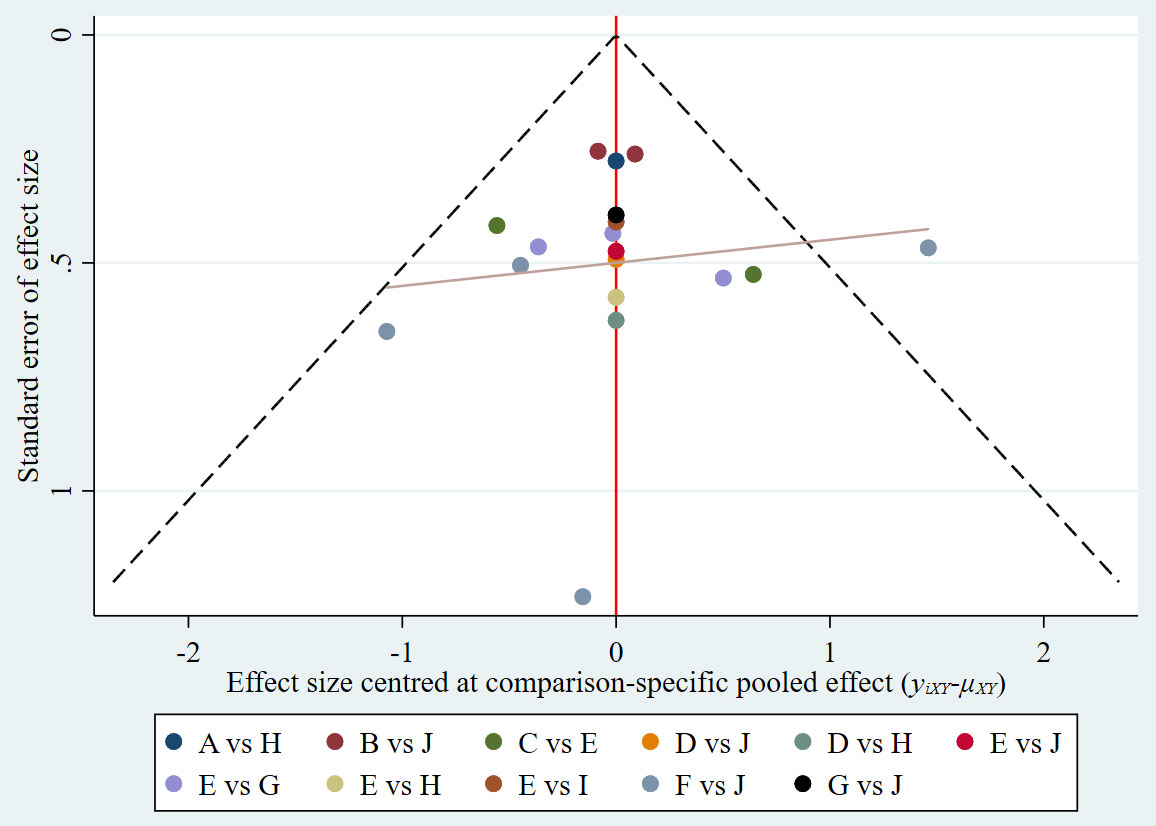
Funnel plot. A: 0. 1 CHG; B: 2% CHG; C: 0. 1% DBDAB; D: 0. 05% Iodophor; E: 0. 5% Iodophor; F: 0. 5% CHG; G: EOW; H: NS; I: Shehuang lotion; J: Sterilized water.

A funnel plot of publication bias is shown in **Figure 5**, and the distribution of each study is basically symmetrical, suggesting no remarkable publication bias.

## 4 Discussion

This analysis examined 10 disinfectants for cleaning the perineum and urethra and preventing catheter-associated urinary tract infections (CAUTI). The evidence network map of different disinfectants for preventing CAUTI shows that the most common disinfectants in this analysis are sterile water and 0.5% iodophor. The forest map and probability ranking showed that the top four disinfectants for the prevention of catheter-related urinary tract infections were chlorhexidine (CHG) 0.5%, snake yellow lotions, acid oxidation potential water (EOW), and iodophor 0.5%.

CAUTIs are associated with increased morbidity, mortality, longer hospital stays, and increased hospitalization costs for patients and the health system. CAUTIs are also associated with a higher risk of antimicrobial resistance (AMR), making it difficult to treat patients. AMR in UTIs is also increasing globally, which further emphasizes the necessity of developing interventions to reduce the incidence of CAUTI (25).

Previous studies or guidelines for CAUTI included catheter materials, such as antibiotic-coated catheters and silver ion catheters. There was also a push for education and training for indwelling catheter operators and those responsible for catheter maintenance. Evidence suggests that reducing bacterial colonization around the urethral area may reduce the risk of CAUTIs (26). Some studies have found that cleaning the perineum with chlorhexidine before an indwelling catheter is placed and reducing bacterial colonization around the urethra may reduce the risk of CAUTI (27). Most of the studies included in this paper compare the effects of cleaning the perineum with two disinfectants on catheter-associated infections. There have been no studies comparing multiple disinfectants at the same time in the hope of finding a safer and more effective disinfectant for preventing catheter-associated infections.

Chlorhexidine (CHG), one of the top four disinfectants found in this analysis (28), is a broad-spectrum and highly effective biguanide that can destroy the plasma membrane of the cell wall of pathogenic microorganisms, thereby rapidly killing pathogens and reducing the number of bacterial colonies on the skin surface, and consequently reducing the risk of infection. CHG is less irritating to the skin and can be more widely applied. CHG is an antimicrobial agent, and long-term use can easily lead to the development of resistance. The high cost will increase the financial burden on patients.

The snake yellow lotion is a kind of Chinese medicine preparation, its main effective components are snake seed, yellow cypress, and matrine. Among them, the snake seed is a commonly used external medicine in traditional Chinese medicine clinical settings, which has the function of sterilization and relieving itching. Phellodendri has a strong inhibitory effect on pyogenic bacteria, especially gram-positive coccus such as Staphylococcus aureus, Epidermococcus, and Streptococcus pyogenes. Matrine has the function of relieving inflammation and detoxifying, killing insects, and relieving itching (15). The results of this analysis showed that washing the perineum with snake yellow lotion can effectively prevent CAUTI, but the relevant literature is limited and there is a need for further research and exploration.

Studies have shown that EOW has a rapid disinfection effect because of chemical disinfection based on physical disinfection, as well as has a strong killing effect on bacterial nutrient morphology and spores (17, 29, 30). EOW can directly destroy cell metabolic enzymes, resulting in bacterial rupture and death, thereby more thoroughly clearing bacteria around the urethral tract (16, 17). After that, EOW can be reduced to ordinary water for a period of time, and as it has no irritating effect on the skin and mucosa of patients, it is safe and does not increase the discomfort of patients. Adverse reactions such as irritating burning pain, redness, and swelling pain are rare, and the irritation produced by EOW is less than that of iodophor (17), so it is an ideal cleaning agent for the perineum and urethral opening.

The top four disinfectants selected in this analysis were 0.5% chlorhexidine (CHG), snake yellow lotion, acidic oxidizing potential water (EOW), and 0.5% iodophor. This analysis provides a variety of possibilities for clinicians to choose the treatment strategy for catheter-associated urinary tract infections.

This analysis was the first to compare multiple disinfectants at the same time, but the definitions of urinary tract infections (that is, based on symptoms or bacterial positive cultures) vary from study to study. The timing of perineal disinfection with the included disinfectants was either before or after the indwelling catheter. Previous studies have mentioned that the type of catheter had a certain contribution to catheter-associated urinary tract infection. Only two papers (13, 15) included in this study mentioned the silicone catheter used, while other studies did not mention the type of catheter used. We expect to further investigate the role of catheter type and perineal skin cleaning before and after catheter indwelling in the prevention of catheter-associated urinary tract infections in future studies.

Conclusion: 0.5% CHG, snake yellow lotions, EOW, and 0.5% iodophor can be used to clean the perineum and urethra in patients with indwelling catheterization, and this action can reduce the risk of CAUTI. However, there are few studies included in this analysis, and further studies are needed to support the effectiveness and safety of 0.5% CHG, snake yellow lotions, EOW, and 0.5% iodoprene in preventing CAUTI.

## Data availability statement

The original contributions presented in the study are included in the article/supplementary material. Further inquiries can be directed to the corresponding author.

## Author contributions

ZS, GZ, CZ, and GX conceptualized the manuscript and edited subsequent versions. ZS, GZ, XM, and WS wrote the first draft. XM, WS, YW, YL, and DW collected and analyzed data. CZ and GX contributed ideas to the texts. All authors approved the final manuscript.
